# Clinical, epidemiological, and mycological features of patients with candidemia: Experience in two tertiary referral centers in Iran

**DOI:** 10.18502/cmm.8.3.11207

**Published:** 2022-09

**Authors:** Mohammad Kord, Mohammadreza Salehi, Seyed Jamal Hashemi, Alireza Abdollahi, Neda Alijani, Ayda Maleki, Shahram Mahmoudi, Kazem Ahmadikia, Nasrin Parsameher, Masoud Moradi, Mahsa Abdorahimi, Sara Rezaie, Shirin Sadat Hashemi Fesharaki, Kiana Abbasi, Laura Alcazar-Fuoli, Sadegh Khodavaisy

**Affiliations:** 1 Department of Medical Parasitology and Mycology, School of Public Health, Tehran University of Medical Sciences, Tehran, Iran; 2 Research center for antibiotic stewardship and antimicrobial resistance, Department of infectious diseases and Tropical Medicine, Imam Khomeini Hospital Complex, Tehran University of Medical Sciences, Tehran, Iran; 3 Department of Pathology, Imam Khomeini Hospital Complex, Tehran University of Medical Sciences, Tehran, Iran; 4 Department of Infectious Diseases, Shariati Hospital, Tehran University of Medical Sciences, Tehran, Iran; 5 Department of Parasitology and Mycology, School of Medicine, Iran University of Medical Sciences, Tehran, Iran; 6 Clinical Laboratory, Shariati Hospital, Tehran University of Medical Sciences, Tehran, Iran; 7 Department of Biostatistics and Epidemiology, Faculty of Health, Mazandaran University of Medical Sciences, Sari, Iran; 8 Department of Microbiology, Shahr-e-Qods Branch, Islamic Azad University, Tehran, Iran; 9 Department of Chemistry and Biology, Ryerson University, Toronto, Canada; 10 Department of Molecular Pathology, Tehran Heart Center, Tehran University of Medical Sciences, Tehran, Iran; 11 Department of Microbiology, Zanjan Branch, Islamic Azad University, Zanjan, Iran; 12 Mycology Reference Laboratory, National Centre for Microbiology, Instituto de Salud Carlos III, Madrid, Spain

**Keywords:** Antifungal susceptibility, Candidemia, Epidemiology, Iran, Risk factors

## Abstract

**Background and Purpose:**

Candidemia is a major cause of morbidity and mortality among patients receiving immunosuppressive therapy and those hospitalized with serious underlying diseases. Here, we investigated the epidemiological, clinical, and mycological features of candidemia in Tehran, Iran.

**Materials and Methods:**

A prospective observational study of all patients diagnosed with candidemia was performed at two referral teaching hospitals in Tehran, Iran, from February to December 2018. Demographic characteristics, underlying diseases, risk factors, clinical symptoms, and laboratory analyses of candidemic patients with positive culture were mined. *Candida* isolates were molecularly identified by sequencing of the internal transcribed spacer region (ITS1-5.8S-ITS2). The antifungal susceptibility testing for fluconazole, itraconazole, voriconazole, posaconazole, amphotericin B, caspofungin, micafungin, and anidulafungin against the isolates was performed using CLSI broth microdilution reference method (M27-A3).

**Results:**

A total of 89 episodes were identified, with an incidence of 2.1 episodes/1000 admissions. The common underling disease were malignancy (46%), renal failure/dialysis (44%), and hypertension (40%). The overall crude mortality was 47%. *C. albicans* (44%) was the most frequent causative agent, followed by *C. glabrata* (21%), *C. parapsilosis* complex (15%), *C. tropicalis* (11%), and *C. lusitaniae* (3.5%). All the isolates were susceptible to amphotericin B. The activity of all four azoles was low against *non-albicans Candida* species, especially *C. tropicalis*.

**Conclusion:**

The increase in non-*albicans Candida* species with reduced susceptibility to antifungal drugs might be alarming in high-risk patients. Therefore, accurate knowledge of predisposing factors and epidemiological patterns in candidemia are effective steps for managing and decreasing the mortality rate in candidemia.

## Introduction

Despite advancements in clinical patient care, *Candida* species remain the most commonly encountered pathogens isolated from bloodstream infections (BSIs) globally and are associated with significant morbidity and mortality, particularly among hospitalized patients receiving immune-suppressive therapy or diagnosed with a serious underlying health condition [ 1 , 2 ]. Depending on the yeast species, the mortality rate may vary from 30% to 85% [ 3 , 4 ]. Although in general, *C. albicans* is still the leading cause of candidemia, a shift towards non-*albicans Candida* (NAC) species has been reported in recent years [ 5 ]. The changing face of candidemia is alarming because NAC species might be associated with increased mortality and antifungal drug resistance [ 6 , 7 ]. Although empirical therapy partly depends on epidemiological data, risk assessment for candidemia, it is critical for clinicians to commence appropriate empirical antifungal therapy [ 8 ]. There are several risk factors associated with candidemia, such as exposure to broad-spectrum antibiotics, surgical procedures, prolonged use of central venous catheters (CVC), dialysis, use of corticosteroids, and cytotoxic chemotherapy [ 9 ]. Again, the diagnosis of candidemia remains a challenging task, despite the development attained in the diagnosis of fungal BSIs during recent years [ 10 - 12 ]. Disparities in the epidemiology of candidemia exist among countries and this seriously influences the need for continuous surveillance to monitor the trend of the disease, the species distribution, and the emergence of antifungal drug resistance [ 13 ]. Although some studies have reported determinants of mortality in candidemia, their results were based on retrospective data and from a restricted viewpoint [ 14 - 17 ]. Accordingly, the reasons for the current poor outcome of candidemia are based on inadequate data. With this idea in mind, we investigated the molecular epidemiology, clinical characteristics, species distribution, antifungal susceptibility profiles, and outcome of candidemia among hospitalized patients in Tehran, the capital of Iran, to provide appropriate perspectives on these patients.

## Materials and Methods

### Study design and patient selection

This study was conducted on hospitalized candidemia patients from February to December 2018 at two tertiary care training centers (Imam Khomeini hospital complex and Shariati hospital) affiliated with Tehran University of Medical Sciences, Tehran, Iran. The studied population included all culture-positive BSI patients irrespective of their age or gender. In this study, we defined nosocomial candidemia as the occurrence of one or more *Candida* species culture-positive blood drawn at least 48 h after admission. We excluded nosocomial candidemia episodes that represented relapses but included fresh episodes that occurred during separate admissions as new cases. The patient’s baseline characteristics, clinical, laboratory, and microbiological data were collected upon confirmation of candidemia. Data were extracted from the patients’ hospital records using a standardized case report form and included the baseline characteristics (age, gender); microbiological parameters (*Candida* species); comorbidities (diabetes mellitus, pulmonary disease, chronic renal failure/hemodialysis, malignancy, cardiovascular diseases, human immunodeficiency virus infection, viral hepatitis, sepsis, and neutropenia [absolute neutrophil count<500 cells/mm^3^]); invasive procedures (including the insertion of a CVC, nasogastric tube, urinary catheterization, immune-suppressive therapy, and intubation) and other risk factors, such as total parenteral nutrition (TPN) within 72 h prior to the onset of candidemia, clinical manifestations, use of broad-spectrum antibiotics and antifungal therapy and outcome parameters - hospital mortality (i.e., death within 30 days of the first documented candidemia episode). In cases where a patient had more than one episode of candidemia, the first episode was used in the risk factor analysis.

### Clinical specimens and identification of Candida species

Blood samples were aseptically obtained from patients with suspected BSI. The samples were inoculated in aerobic blood culture medium bottles (BacT/ALERT® Culture Media/bioMerieux) and incubated within the automated Bactecsystem (BACT/ALERT® 3D). Through observation, we identified and then subculture the initial positive blood cultures onto Sabouraud dextrose agar (SDA) supplemented with 0.5% chloramphenicol and incubated at 37ºC for 24h to 48h. Yeast-like colonies were sub-cultured on CHROMagar *Candida* medium (CHROMagar Company, Paris, France) to ensure purity, and then identified using the automated Vitek 2 YST ID Card system (bioMérieux, Marcy-L’Etoile, France),according to the manufacturer’s instructions [ 18 ]. Molecular identification was conducted for all recovered isolates. Briefly, we extracted the genomic DNA from cultures grown on SDA using the Genomic Extraction Kit (GeneAll, Korea), according to the manufacturer’s instructions and stored it at-20°C till the next use. The internal transcribed spacer rDNA region (ITS1-5.8S-ITS2)was amplified and sequenced using ITS1 and ITS4 primers, as previously described by Leaw *et al*. [ 19 ].Thereafter, we performed a bidirectional chain-terminated Sanger sequencing with the same primers used for the amplification. We processed the sequence data using the Lasergene-SeqMan software (version 9.0.4, DNASTAR) and aligned the results with the data in the GenBank database (https://blast.ncbi.nlm.nih.gov) and the Westerdijk Fungal Biodiversity Institute (Utrecht, The Netherlands) research database (http://www.westerdijkinstitute.nl/). Identification was defined by > 99.5% sequence similarity, with ≥ 95% query coverage.

### Antifungal susceptibility testing

Antifungal susceptibility testing was performed according to the Clinical and Laboratory Standards Institute broth microdilution guidelines (CLSI-M27-A3 and M60) [ 20 , 21 ]. All tests were performed in duplicate, on two different days. *C. krusei* (ATCC 6258) and *C. parapsilosis* (ATCC 22019) were used as quality control strains, as recommended by the CLSI. Data interpretation was based on clinical break points and epidemiological cut-off values [ 21 , 22 ].

### Statistical analyses

The SPSS software (version 22.0; SPSS Inc., Chicago, IL, USA) was used for statistical analysis in this study. The median, mean, standard deviation (SD), maximum, and minimum values were utilized to describe quantitative data and categorical data were described using frequencies. The Chi-square test or Fisher’s exact test and the Student’s t-test were employed to evaluate categorical and continuous variables, respectively. Logistic regression analyses were performed to identify independent variables associated with candidemia due to *C. albicans* and non-*C.albicans* spp. and the final outcome.

## Results

### Patient characteristics and risk factors

In total, 89 patients with candidemia among 41,540 hospitalized patients were enrolled in this study. The demographic characteristics, clinical manifestations, types of the underlying disorder, comorbidities increasing risk of candidemia, medications, and outcome of the disease are summarized in Table 1. Totally, the prevalence of candidemia in the present study was 2.1 per 1000 hospital admissions. The patient’s age ranged from 21 days to 93 years, with a mean age of 49.6 years. The majority of patients (61/89; 68.5%) were over 40 years of age, and only six patients were under 16 years. It should be noted that 42 patients (42/89; 47.2%) were male. The prevalence of candidemia in various hospitalized wards was as follows: 39.3% in the intensive care units (ICUs), 18.0% in hemato/oncology wards, 10.1% in the internal ward, 5.6% in the surgical ward, and 6.7%in the kidney/urology unit. The most common underlying disease were malignancy (41/89; 46.1%), renal failure/dialysis (39/89; 43.8%), hypertension (HTN) (32/89; 40.0%), and lung disorders (28/89; 31.5%). The majority of patients had multiple risk factors. The most common risk factors for candidemia in the present study were CVC (67/98; 75.3%), mechanical ventilation (49/89; 52.8%), and urinary catheterization (46/89; 51.7%). Fever and sepsis were the most frequent clinical manifestation of candidemia (59/89; 66.3%), followed by diarrhea (27/89; 30.3%), cough (22/89; 24.7%), chills (20/89; 22.5%), and pleural effusion (16/89; 18.0%). 

**Table 1:** Demographic characteristics, clinical manifestations, risk factors, medications, and outcome of patients with candidemia

Characteristics	All cases (n=89)	*C. albicans* (n=39)	*C. glabrata* (n= 19)	*C. parapsilosis* (n= 14)	*C. tropicalis* (n= 10)	*C. lusitaniae* (n=3 )	*C. kefyr* (n=1 )	*C. guilliermondii* (n=1 )	*C. krusei* (n=1 )	*C. dubliniensis* (n=1 )
Demographic										
<1 year	3 (3.4)	2 (5.1)	1 (5.3)	0	0	0	0	0	0	0
1-15 years	3 (3.4)	1 (2.5)	1 (5.3)	1 (7.1)	0	0	0	0	0	0
16-40 years	22 (24.7)	10 (25.6)	0	6 (42.9)	4 (40.0)	0	1 (100)	1 (100)	0	0
41-60 years	36 (40.4)	17 (43.6)	8 (42.1)	4 (28.6)	4 (40.0)	2 (66.7)	0	0	1 (100)	0
>60 years	25 (28.1)	9 (23.1)	9 (47.4)	3 (21.4)	2 (20.0)	1 (33.3)	0	0	0	1 (100)
Median age, years (range)	49.6 (1-93)	47.8 (1-86)	58.8 (1-88)	40.5 (14-79)	50.3 (27-89)	65.3 (50-93)	29	16	53	65
Gender; male (%)	42 (47.2)	22 (56.4)	7 (36.8)	6 (42.9)	5 (50)	0	1 (100)	0	1 (100)	0
Median days in hospital (range)	42 (1-347)	41 (1-347)	42.6 (5-126)	89.1 (7-192)	50.9 (1-130)	46 (31-54)	197	101	30	4
Hospital Ward										
ICUs	35 (39.3)	16 (41.0)	7 (36.8)	6 (42.9)	3 (30.0)	2 (66.7)	1 (100)	0	0	0
Hematology and oncology	16 (18)	4 (10.3)	4 (21.1)	3 (21.4)	2 (20.0)	1 (33.3)	0	0	1 (100)	1 (100)
Medical	9 (10.1)	2 (5.1)	2 (10.5)	2 (14.3)	2 (20.0)	0	0	1 (100)	0	0
Surgical	5 (5.6)	3 (7.7)	0	1 (7.1)	1 (10.0)	0	0	0	0	0
Kidney/Urology	6 (6.7)	3 (7.7)	3 (15.8)	0	0	0	0	0	0	0
Infectious	5 (5.6)	2 (5.1)	1 (5.3)	1 (7.1)	1 (10.0)	0	0	0	0	0
Other	13 (14.6)	9 (23.1)	2 (10.5)	1 (7.1)	1 (10.0)	0	0	0	0	0
Underlying disease										
DM (%)	27 (30.3)	11 (28.2)	8 (42.1)	3 (21.4)	2 (20.0)	2 (66.7)	0	0	1 (100)	0
Malignancy (%)	41 (46.1)	11 (28.2)	11 (57.9)	8 (57.1)	7 (70.0)	1 (33.3)	0	1 (100)	1 (100)	1 (100)
HTN	32 (40.0)	15 (38.5)	10 (52.6)	3 (21.4)	2 (20.0)	2 (66.7)	0	0	0	0
HIV infection (%)	3 (3.4)	2 (5.1)	0	1 (7.1)	0	0	0	0	0	0
Viral Hepatitis (%)	6 (6.7)	4 (10.3)	1 (5.3)	1 (7.1)	0	0	0	0	0	0
Transplant (%)	6 (6.7)	3 (7.7)	1 (5.3)	2 (14.2)	0	0	0	0	0	0
Vascular and heart events (%)	26 (29.1)	10 (25.6)	9 (47.4)	4 (28.4)	2 (20.0)	1 (33.3)	0	0	0	0
Renal failure/ dialysis (%)	39 (43.8)	20 (51.3)	9 (47.4)	4 (28.6)	6 (60.0)	0	0	0	0	0
Pneumonia/Lung diseases (%)	28 (31.5)	16 (41.0)	3 (15.8)	4 (28.4)	2 (20.0)	2 (66.7)	0	0	0	1 (100)
Risk factors										
CVC (%)	67 (75.3)	28 (71.8)	15 (78.9)	11 (78.6)	6 (60.0)	3 (100)	1 (100)	1 (100)	1 (100)	1 (100)
Urinary catheterization (%)	46 (51.7)	18 (43.9)	11 (57.9)	9 (64.3)	4 (40.0)	2 (66.7)	1 (100)	0	1(100)	
Total parenteral nutrition (%)	10(11.2)	4 (9.8)	3 (15.8)	3 (21.4)	0	0	0	0	0	0
Immunosuppressive (%) therapy	22 (24.7)	9 (22)	7 (31.6)	3 (35.7)	2 (20.0)	0	0	0	1 (100)	0
Neutropenia (%)	15 (16.9)	4 (9.8)	3 (15.8)	5 (35.7)	2 (20.0)	0	0	1 (100)	0	0
Mechanical ventilation (%)	47 (52.8)	19 (48.7)	10 (52.6)	8 (57.1)	6 (60.0)	2 (66.7)	1 (100)	0	0	1 (100)
Clinical manifestations										
Fever	59 (66.3)	29 (70.7)	12 (63.2)	8 (57.1)	5 (50.0)	3 (100)	0	1 (100)	1 (100)	0
Diarrhea	27 (30.3)	12 (29.3)	6 (31.6)	5 (35.7)	3 (30.0)	1 (33.3)	0	0	0	0
Cough	22 (24.7)	13 (31.7)	4 (21.1)	3 (21.4)	2 (20.0)	0	0	0	0	0
Chills	20 (22.5)	10 (24.4)	4 (21.1)	5 (35.7)	1 (10.0)	0	0	0	0	0
Respiratory symptoms	16 (18.0)	6 (14.6)	2 (10.5)	4 (28.6)	2 (20.0)	1 (33.3)	1 (100)	0	0	0
Abdominal infections	11 (12.4)	6 (14.6)	3 (15.8)	1 (7.1)	1 (10.0)	0	0	0	0	0
Dysuria	11 (12.4)	6 (14.6)	2 (10.5)	3 (21.4)	0	0	0	0	0	0
Ascites	11 (12.4)	6 (14.6)	3 (15.8)	1 (7.1)	0	1 (33.3)	0	0	0	0
Seizures	7 (7.9)	2 (4.9)	2 (10.5)	2 (14.3)	1 (10.0)	0	0	0	0	0
Medication										
Broad spectrum antibiotic	82 (92.1)	35 (89.7)	19 (100)	14 (100)	8 (80.0)	3 (100)	1 (100)	0	1 (100)	1 (100)
Corticosteroid	22 (24.7)	9 (23.1)	6 (31.6)	4 (28.6)	2 (20.0)	0	0	0	1 (100)	0
Fluconazole	33 (37.1)	13 (33.3)	7 (36.8)	8 (57.1)	3 (30.0)	0	0	0	1 (100)	1 (100)
Amphotericin B	22 (24.7)	7 (17.9)	4 (21.0)	8 (57.1)	2 (20.0)	0	0	0	1 (100)	0
Caspofungin	29 (32.6)	9 (23.0)	10 (52.6)	7 (50.0)	2 (20.0)	1 (33.3)	0	0	0	0
Nystatin	3 (3.4)	1 (2.6)	1 (5.2)	1 (7.1)	0	0	0	0	0	0
Voriconazole	7 (7.7)	3 (7.7)	1 (5.2)	3 (21.4)	0	0	0	0	0	0
Outcome										
Died	42 (47.2)	19 (48.7)	9 (47.4)	5 (35.7)	6 (60.0)	2 (66.7)	1 (100)	0	0	0
Survived	47 (52.8)	20 (51.3)	10 (52.6)	9 (64.3)	4 (40.0)	1 (33.3)	0	1 (100)	1(100)	1 (100)

CVC: central vein catheter

### Distribution of Candida species

In the study, 89 *Candida* isolates were recovered as agents of candidemia in culture-positive blood from patients. The leading agents of nosocomial candidemia were *C. albicans* (39/89; 43.8%), followed by *C. glabrata* (19/89; 21.3%), *C. parapsilosis* complex (14/89; 15.7%), *C. tropicalis* (10/89; 11.2%), *C. lusitaniae* (3/89; 3.4%), and other species (*C. dubliniensis*, *C. kefyr*, *C. krusei*, and *C. guilliermondii*) (4/89; 4.4%).Table 1 illustrates the distribution of *Candida* species per patients’ age categories. *C. albicans*, *C. glabrata*, and *C. parapsilosis* were the most common species in the age range of 41-60 years (17/39; 43.6%), >60 years (9/19; 47.4%), and 16-40 years (6/14; 42.9%), respectively. The common *Candida* species [*C. albicans* (16/39; 41.0%), *C. glabrata* (7/19; 36.8%), *C. parapsilosis* complex (6/14; 42.9%), and *C. tropicalis* (3/10; 30%)] were more frequently isolated from ICU. Based on *Candida* species, fever (29/39; 74.4%) and cough (13/39; 33.3%) were more prevalent in *C. albicans* infections, while diarrhea was more prevalent in *C. glabrata* (6/19; 31.6%) and *C. parapsilosis* complex (5/14; 35.7%), and pleural effusion was more observed in *C. parapsilosis* infections (4/14; 28.6%). The most common underlying disease in *C. glabrata*, *C. parapsilosis* complex, and *C. tropicalis* infections were renal failure/dialysis (20/39; 51.3%), malignancy (11/19; 57.9%) and sepsis (10/14; 71.4%), as well as malignancy (7/10; 70%), respectively. CVC was the most common risk factor of candidemia due to *C. albicans* (28/39; 71.8%), *C. glabrata* (15/19; 78.9%), *C. parapsilosis* (11/14; 78.6), and *C. tropicalis* (6/10; 60%).Table 2 depicts the multivariate analysis of risk factors for candidemia due to *C. albicans* and NAC species. This analysis revealed that malignancy was an independent risk factor for candidemia (*P=0.013*).

**Table 2:** Multivariate analysis of risk factors for candidemia due to Candida albicans and non-albicans Candida

Characteristics	Population data (n=89)			
	*C. albicans* (n=39)	Non-*albicans Candida* (n=50)	OR (95% Cl)	*P-value*
Gender; male	22	20	0.62 (0.27,1.43)	0.26
Mean age, years (range)	47.8 (1-86)	50.9 (1-93)	1.01 (0.99 , 1.02)	0.59
Underlying diseases				
DM	11	16	1.36 (0.55 , 3.41)	0.51
Malignancy	11	30	3.01 (1.26 , 7.22)	0.013
HIV infection	2	1	0.42 (0.04 , 4.75)	0.48
Viral Hepatitis	4	2	0.40 (0.07 , 2.32)	0.31
Transplant	3	3	0.84 (0.16 , 4.43)	0.84
Vascular and heart events	10	16	1.55 (0.61 , 3.94)	0.36
Renal failure/ dialysis	20	19	0.62 (0.22 , 1.76)	0.37
Pneumonia/Lung diseases	16	12	0.52 (0.21 , 1.29)	0.16
CVC	28	39	1.23 (0.47 , 3.24)	0.67
Urinary catheterization	18	28	1.79 (0.77 , 4.15)	0.18
Total Parenteral nutrition	4	6	1.32 (0.35 , 5.05)	0.68
Immunosuppressive therapy	9	13	1.32 (0.50 , 3.50)	0.58
Neutropenia	4	11	2.75 (0.80 , 9.42)	0.11
Intubation	19	28	1.62 (0.70 , 3.76)	0.26

Abbreviations: DM: diabetes mellitus, CVC: central vein catheter

### Treatment and outcome

The analysis of patients with candidemia showed that either selection of antifungal drugs or the duration of antifungal therapy was inadequate. A total of 60 episodes of candidemia (60/89; 67.4%) received antifungal therapy. Initial treatment was with fluconazole in 33 patients (33/89; 37.1%), caspofungin in 29 patients (29/89; 32.6%), liposomal amphotericin B in 22 patients (22/89; 24.7%), and 14 patients (14/89; 15.7%) received other antifungal agents (e.g., voriconazole) (Table 1). Meanwhile, 29 patients (29/89; 32.6%) did not receive any antifungal drug out of which 19 (19/29; 65.5%) patients expired. The crude mortality rate among 89 patients with candidemia was 47.2% (42/89). Mortality was similar among those infected with *C. albicans* (19/39; 48.7%) and *C. glabrata* (9/19; 47.4%), but lower in patients with *C. parapsilosis* (5/14; 35.7%) than other species. In the multivariate analyses of risk factors for BSIs mortality, intubation (*P=0.001*) and urinary catheterization (*P=0.03*) were independent risk factors for mortality (Table 3).

**Table 3:** Risk factors for 30-day mortality due to candidemia

Characteristics	30-day outcome			Logistic Regression analysis	
	Survival (n=47)	Death (n=42)	*P-value*	OR (95% Cl)	*P-value*
Gender, male (n, %)	24 (51.1)	18 (42.8)	0.44	0.72 (0.31 , 1.66)	0.44
Age (years, Mean ±SD)	45.43±25.39	54.24 ±17.92	0.06	1.02 (0.99 , 1.04)	0.07
Underlying diseases					
DM	15 (31.9)	12 (28.6)	0.73	0.85 (0.34 , 2.11)	0.73
Malignancy	19 (40.4)	22 (52.4)	0.26	1.62 (0.70 , 3.76)	0.26
Viral Hepatitis	1 (2.1)	5 (11.9)	0.10	6.22 (0.70 , 55.56)	0.10
Transplant	3 (6.4)	3 (7.1)	>0.99	1.13 (0.22 , 5.92)	0.89
Vascular and heart events	14 (29.8)	12 (28.6)	0.90	0.94 (0.38 , 2.36)	0.90
Renal failure/ dialysis	16 (34.0)	23 (54.8)	0.79	0.87 (0.31 , 2.46)	0.79
Pneumonia/Lung diseases	12 (25.3)	16 (38.1)	0.20	1.80 (0.73 , 4.43)	0.21
Risk factors					
CVC	32 (68.1)	35 (83.3)	0.10	2.34 (0.85 , 6.48)	0.10
Urinary catheterization	19 (40.4)	27 (64.3)	0.03	2.65 (1.12 , 6.26)	0.03
Total Parenteral nutrition	5 (10.6)	5 (11.9)	>0.99	1.14 (0.30 , 4.23)	0.85
Immunosuppressive therapy	12 (25.5)	10 (23.8)	0.85	0.91 (0.35 , 2.40)	0.85
Neutropenia	11 (23.4)	4 (9.5)	0.08	0.34 (0.10 , 1.18)	0.09
Mechanical ventilation	18 (38.3)	31 (73.8)	0.001	4.41 (1.80 , 10.80)	0.001

Abbreviations: DM: diabetes mellitus, CVC: central vein catheter

### Antifungal susceptibility testing

Table 4 depicts the MIC ranges, MIC_50_, MIC_90_, geometric means (GM) MIC, and MIC modes of eight antifungal drugs against 89 *Candida* isolates recovered from candidemic patients. In terms of MIC_50_ and MIC_90_, echinocandins demonstrated the highest MIC against *C. parapsilosis* (1μg/ml and 4μg/ml for caspofungin, 2μg/ml for micafungin, 2μg/ml and 4μg/ml for anidulafungin) which was higher than other NAC species. However, we did not detect resistance to echinocandins in any of the *C. tropicalis*, *C. guilliermondii*, *C. lusitaniae*, *C. krusei*, and *C. kefyr* isolates. Among common species, *C. albicans* showed high susceptibilities to fluconazole (97.4%), while fluconazole susceptibility was lower in NAC species, particularly in *C. tropicalis* (S=40%). 

**Table 4:** *In vitro* activities of eight antifungal agents again *Candida* species isolated from 89 patients with candidemia

Species (n)	Antifungal	MIC Range (μg/ml)	MIC_50_	MIC_90_	GM	Mode	% S/WT*
*C. albicans* (39)	FLU	0.25-32	0.5	1	0.496	0.5	97.4
	ITR	0.016-1	0.06	0.25	0.080	0.06	92.3
	VRC	0.008-1	0.015	0.03	0.014	0.008	97.4
	AMB	0.25-1	0.25	1	0.359	0.25	100
	CAS	0.03-8	0.06	0.25	0.088	0.06	94.8
	MFG	0.008-4	0.03	0.125	0.037	0.08	94.8
	AFG	0.015-1	0.12	0.125	0.091	0.125	94.8
	PSZ	0.015-1	0.03	0.125	0.034	0.03	97.4
*C. glabrata* (19)	FLU	1-64	16	32	14.87	32	0
	ITR	0.12-4	1	4	0.862	1	100
	VRC	0.03-2	0.5	1	0.499	1	94.7
	AMB	0.25-1	0.5	1	0.540	0.5	100
	CAS	0.03-1	0.125	1	0.157	0.12	73.7
	MFG	0.007-0.5	0.16	0.16	0.051	0.15	94.7
	AFG	0.03-1	0.06	0.125	0.060	0.03	94.7
	PSZ	0.06-2	1	2	0.829	1	84.2
*C. parapsilosis* (14)	FLU	0.25-64	1	32	1.104	1	78.6
	ITR	0.06-2	0.125	1	0.191	0.125	100
	VRC	0.015-0.25	0.25	0.25	0.035	0.016	85.7
	AMB	0.06-1	0.25	1	0.335	0.25	100
	CAS	0.25-8	1	6	1.16	1	78.6
	MFG	0.5-8	2	5	1.485	2	92.9
	AFG	1-8	2	6	1.811	2	92.9
	PSZ	0.06-1	0.06	0.5	0.099	0.06	85.7
*C. tropicalis* (10)	FLU	1-64	4	64	6.96	ND	40
	ITR	0.25-16	0.5	16	1.31	0.25	50
	VRC	0.12-16	0.5	16	0.995	ND	20
	AMB	0.5-2	0.5	2	0.707	0.5	100
	CAS	0.03-0.25	0.125	0.25	0.106	ND	100
	MFG	0.007-0.06	0.03	0.06	0.025	0.03	100
	AFG	0.015-0.12	0.06	0.125	0.068	0.06	100
	PSZ	0.12-1	0.5	1	0.375	0.5	40
*C. lusitaniae* (3)	FLU	1-4	1	ND	1.58	ND	100
	ITR	0.12-1	0.5	ND	0.416	ND	100
	VRC	0.015-0.25	0.016	ND	0.038	ND	100
	AMB	0.5-1	0.5	ND	0.629	0.5	100
	CAS	0.25-0.5	0.5	ND	0.396	0.5	100
	MFG	0.12-0.25	0.25	ND	0.195	0.25	100
	AFG	0.125-0.25	0.25	ND	0.198	0.25	100
	PSZ	0.03-0.12	0.03	ND	0.075	0.125	33.3
*C. guilliermondii* (1)	FLU	2	ND	ND	ND	ND	100
	ITR	0.25	ND	ND	ND	ND	100
	VRC	0.06	ND	ND	ND	ND	100
	AMB	0.25	ND	ND	ND	ND	100
	CAS	0.25	ND	ND	ND	ND	100
	MFG	0.5	ND	ND	ND	ND	100
	AFG	0.5	ND	ND	ND	ND	100
	PSZ	0.25	ND	ND	ND	ND	100
*C. dubliniensis* (1)	FLU	0.25	ND	ND	ND	ND	100
	ITR	0.016	ND	ND	ND	ND	100
	VRC	0.016	ND	ND	ND	ND	100
	AMB	0.125	ND	ND	ND	ND	100
	CAS	0.06	ND	ND	ND	ND	100
	MFG	0.008	ND	ND	ND	ND	100
	AFG	0.12	ND	ND	ND	ND	100
	PSZ	0.03	ND	ND	ND	ND	100
*C. krusei* (1)	FLU	8	ND	ND	ND	ND	100
	ITR	1	ND	ND	ND	ND	100
	VRC	1	ND	ND	ND	ND	100
	AMB	1	ND	ND	ND	ND	100
	CAS	0.5	ND	ND	ND	ND	100
	MFG	0.125	ND	ND	ND	ND	100
	AFG	0.06	ND	ND	ND	ND	100
	PSZ	0.125	ND	ND	ND	ND	100
*C. kefyr* (1)	FLU	0.5	ND	ND	ND	ND	100
	ITR	0.25	ND	ND	ND	ND	100
	VRC	0.06	ND	ND	ND	ND	100
	AMB	0.5	ND	ND	ND	ND	100
	CAS	2	ND	ND	ND	ND	100
	MFG	0.5	ND	ND	ND	ND	100
	AFG	0.5	ND	ND	ND	ND	100
	PSZ	0.016	ND	ND	ND	ND	100

Abbreviations: GM, geometric mean; S: susceptible; WT: wild-type; AMB: amphotericin B; FLU: fluconazole; ITR: itraconazole; VRC: voriconazole; PSZ: posaconazole; AFG: anidulafungin; MFG: micafungin.

* Percentage of susceptible wild-type isolates based on the clinical breakpoint values or epidemiological cutoff values.

Accordingly, *C. glabrata* exhibited the highest MIC_50_ for fluconazole (16μg/ml). The activity of all four azoles was low against NAC species; however, *C. albicans* has a lower MIC_50_ (0.5, 0.06, 0.015, and 0.03 μg/ml) and MIC_90_ (1, 0.25, 0.03, and 0.12μg/ml) for fluconazole, itraconazole, voriconazole, and posaconazole, respectively.

## Discussion

We focused on candidemia patients and found that *C. albicans* was the most prevalent of the candidemia episodes (43.8%). Among the NAC species, *C. glabrata* was the predominant species, followed by *C. parapsilosis*, *C. tropicalis*, and *C. lusitaniae*. In most previous studies across the world, *C. albicans* was the most common species isolated from candidemia, which is consistent with the results of our study [ 13 ]. Despite this, in a systematic review, *C. parapsilosis* (30.8%) was the leading agent of candidemia in Iran [ 23 ]. Similar to previous studies, *C. glabrata* is the most common NAC species in this study [ 24 - 26 ]. However, in other studies, *C. parapsilosis* has been the most prevalent NAC species [ 27 - 30 ]. Moreover, the increasing mortality rate associated with the increased frequency of NAC might be linked to the high rate of treatment failure resulting from either acquired or intrinsic resistance to the few antifungal drugs available to manage candidemia. For example, echinocandins are regarded as the first-line drug for the treatment of *C. glabrata* BSI. However, the increasing reports on refractory BSI caused by fluconazole and echinocandins resistant *C. glabrata* isolates are alarming. It has been shown that *C. lusitaniae* can rapidly acquire multidrug resistance traits (MDR) during the course of antifungal treatment with fluconazole, amphotericin B, and caspofungin [ 31 ]. *C. kefyr* can cause serious infection in patients with hematologic malignancies and recently has shown resistance to amphotericin B [ 32 ]. *C. norvegensis* is also shown to be azole-resistant [ 33 ]. While 20% of the NAC species in this study were fluconazole-resistant, only 2.6% of the *C. albicans* species were resistant to fluconazole. Our study confirmed that primary fluconazole resistance is uncommon in *C. albicans*. The majority of *C. albicans* are sensitive to amphotericin B, fluconazole, itraconazole, voriconazole, posaconazole, and echinocandins in vitro, especially in patients without a history of exposure to antifungal agents [ 34 ]. A recent study looked at the impact of the new CLSI breakpoints and demonstrated that applying revised fluconazole breakpoints increased the rate of fluconazole resistance in *C. albicans*, *C. tropicalis*, and *C. parapsilosis* [ 35 ]. The occurrence of fluconazole resistance in *C. tropicalis* has been previously reported as 5.0%–7.2% from two reports from the ARTEMIS study over 12 years [ 36 , 37 ]. The high rate of azole non-susceptible *C. tropicalis* in this study was similar to other studies from Asia [ 38 , 39 ]. Moreover, among the fluconazole-resistant *C. tropicalis* isolates in our study, six were resistant to voriconazole. Many candidemia studies revealed a significant increase in azole-resistant *C. tropicalis* blood isolates and some reported pan-azole and amphotericin B-resistant isolates [ 40 ]. An extensive candidemia study in India showed that *C. tropicalis* and *C. auris* isolates also carry the MDR traits [ 41 ]. The susceptible dose-dependent (SDD) *C. glabrata* isolate is defined as fluconazole MIC ≤ 32 mg/l since 2012. Overall, 94.7% of *C. glabrata* isolates were categorized as SDD (MIC_50_= 16mg/l, MIC_90_= 32 mg/l) that is consistent with the findings of studies from the Asian-Pacific region [ 42 ]. As demonstrated in the current study, no resistance to any of the antifungal agents was observed in *C. guilliermondii*, *C. lusitaniae*, *C. kefyr*, and *C. krusei* (except for the intrinsic fluconazole resistance in *C. krusei*). Furthermore, resistance to echinocandins was very low, except for *C. parapsilosis*, which exhibited higher MICs than those of other *Candida* species. 

The increased frequency of NAC may also be attributable to the improved diagnostic technique, allowing NAC species to be characterized with methods that are more sensitive. In this study, all *Candida* isolates were identified using the DNA sequencing method to assess the exact epidemiological pattern of species distribution. The mean age of the patients in this study was 49.6 years. Most of these patients were over 40years old (68.5%). The mean age in other studies varies from 40 to 65 years. Candidemia patients (usually with underlying conditions, such as diabetes, cancer, as well as pulmonary and heart complications) mostly are admitted to ICUs. The overall crude mortality of 47.2% in our study is similar to that reported by other investigators from Iran [ 43 - 45 ] but considerably higher than the 26% quoted by Chen *et al*. [ 46 ].Totally, 60patients took at least one antifungal drug, and the mortality rate for these patients was 38.3%(23/60; 38.3%). However, the mortality rate among patients who did not receive antifungal drugs was 65.5% (19/29; 65.5%). This suggests that early diagnosis and timely antifungal administration can dramatically reduce the rate of mortality. The highest rate of mortality was found among ICU patients (65.7%) which is unsurprising, given the severity of underlying illness in this population. Evidence supports the fact that patients admitted to the ICU have higher mortality rates than those in other wards[ 47 ].The major underlying diseases were malignancy, sepsis, renal failure/dialysis, and HTN. Diabetes mellitus, cardiovascular diseases, and pulmonary disorders were other underlying diseases. Consistently, similar underlying conditions were documented in studies conducted in Turkey [ 48 ], China [ 9 ], and Australia [ 49 ]. Multivariate analyses of risk factors for BSIs caused by *C. albicans* and NAC species showed that malignancy was an independent risk factor for candidemia (*P=0.013*). In the multivariate analyses of risk factors for BSIs mortality, intubation (*P=0.001*) and urinary catheterization (*P=0.03*) were independent risk factors for mortality. In a prospective study performed in the ICU of a tertiary care hospital in Athens, the authors noted that the administration of glucocorticoids, presence of CVCs, and candiduria were independent risk factors for candidemia caused by NAC species [ 50 ]. Although neutropenia and total parenteral nutrition are well-known risk factors attributed to mortality in candidemia patients[ 51 ], no significant correlation was observed in the present study. The current study had some limitations. First, in this study, two main medical centers in the capital of Iran had been selected; however, information from other centers has not been included in this study to obtain insights into the epidemiological status of candidemia in Tehran. Second, the results of the multivariate analyses might be influenced by the sample size and the number of variables included in the models.

## Conclusion

Candidemia with a shift in species distribution towards NAC species remains a lethal disease. The results of this study provide important information regarding the distribution of *Candida* species in patients with candidemia in Tehran, the capital of Iran, for which there is a paucity of data regarding the epidemiology , risk factors, and antifungal susceptibility patterns of these species. Accurate knowledge of predisposing factors and epidemiological patterns can be an effective step in disease management. In this study, *C. albicans* is reported to be the most common species causing candidemia; however, an increasing frequency of NAC species could pose a serious therapeutic challenge due to different antifungal susceptibility patterns. This report shows that candidemia is a significant source of morbidity in Tehran.

Ethical approval

Before the collection of samples, the Ethics Committee of Tehran University of Medical Science, Tehran, Iran (IR.TUMS.SPH.REC.1396.4195) approved the procedures to be used in this study. In addition, in line with the principles of research ethics, written informed consent was obtained from individual patients.

## Acknowledgments

The authors express their gratitude to their colleagues, Mohammad Reza Safari and Mahsa Domanlo, for their assistance in conducting this study.

## Authors’ contribution

S.KH., M.K., S.J.H., and M.R.S. designed the study. Material preparation, data collection, and analysis were performed by S.K.H., M.K., S.R., K.A., M.A., SH.M., A.M., M.M., N.P., K.A., N.A., and SH.S.H. The first draft of the manuscript was written by M.K., A.A., L.A.F, and S.R., and all authors commented on previous versions of the manuscript. All authors read and approved the final manuscript. 

## Conflicts of interest

The authors have no conflicts of interest to declare that are relevant to the content of this article.

## Financial disclosure

This study has been funded and supported by Tehran University of Medical Sciences, Tehran, Iran (Grant no. 99-2-99-48944).
